# Editorial: Innovative flame retardant materials: balancing efficiency, safety, and sustainability

**DOI:** 10.3389/fchem.2026.1788165

**Published:** 2026-01-21

**Authors:** Fei Xiao, Siqi Huo, Zongmin Zhu, Xiaodong Jin

**Affiliations:** 1 School of Safety Science and Emergency Management, Wuhan University of Technology, Wuhan, China; 2 School of Engineering, Centre for Future Materials, University of Southern Queensland, Springfield Central, Toowoomba, QLD, Australia; 3 College of Materials Science and Engineering, Hubei Key Laboratory for New Textile Materials and Applications, Wuhan Textile University, Wuhan, China; 4 College of Materials Science and Engineering, Beijing University of Technology, Beijing, China

**Keywords:** composites, flame retardant, polymer, synergy, thermal stabilily

As the global industrial landscape shifts toward high-performance polymeric materials in sectors such as electric vehicle (EV) manufacturing, 5G telecommunications, and aerospace, the demand for advanced fire retardancy has become increasingly critical (Wang et al., 2023; Liu et al., 2022). Historically, scientific approaches to fire hazards have evolved from rudimentary topical treatments to the sophisticated macromolecular engineering in the twentieth century (Liu et al., 2022). However, the modern challenge extends beyond merely achieving fire resistance; it requires balancing overall performance while adhering to environmental stewardship principles and ensuring human safety. For decades, organo-halogen compounds dominated the market due to their exceptional gas-phase efficiency and low production costs. The recognition that these substances are persistent, bioaccumulative, and toxic has triggered a regulatory revolution (Kemmlein et al., 2009). This Research Topic serves as a nexus for studies addressing these challenges, exploring the intricate chemistry of synthesis, combustion mechanisms, and the development of sustainable alternatives that meet stringent safety standards without compromising material performance. The four invited articles in this Research Topic highlight recent advances in fire retardancy research, encompassing relevant aspects of the current landscape.

The first article by Howell focuses on the promising future of sustainable flame retardants. It envisions future flame retardants—whether biobased, organophosphorus, reactive, or oligomeric—and proposes that these novel flame retardants must meet criteria of effectiveness, environmental friendliness, and sustainable production. These materials should be producible through simple, low-cost processes, exhibit broad compatibility with various polymer matrices, and be easily degradable during polymer recycling. Most importantly, they must be non-migratory and non-toxic.

The second article by Dong et al. is dedicated to the pyrolysis characteristics of polyurethane (PU) using reactive force field molecular dynamics simulations to elucidate product distribution and thermal decomposition mechanisms. A molecular model of polyurethane was constructed, and its pyrolysis process was simulated at temperatures ranging from 1,500 to 3,000 K. The study analyzed potential energy changes, product species, the distribution of carbon-containing components, main gaseous products, primary intermediate products, and initial cleavage pathways. This work provides microscopic insights into the thermal degradation of polyurethane, supporting applications in fire safety assessment and material recycling.

The third article by Wu et al. discusses the co-pyrolysis and combustion characteristics of polylactic acid (PLA) and acrylonitrile-butadiene-styrene (ABS). The results demonstrate that PLA can catalyze the pyrolysis of ABS in the blend, leading to a more vigorous combustion reaction. The presence of ABS enhances the thermal degradation of PLA, exhibiting a significant synergistic effect on both the heat release rate and total heat release. Kinetic analysis indicates that the interaction between PLA and ABS accelerates the degradation process, particularly at lower temperatures. These findings are highly significant for the efficient utilization and fire-retardant properties of PLA/ABS blends.

Metal-organic framework (MOF)-derived flame retardants have recently emerged as innovative additives in polymer materials, effectively reducing fire risks in applications with stringent fire safety requirements (Zhang et al., 2020). The fourth article by Aseeri et al. reports the fabrication of a novel hybrid hydrogel incorporating a scandium-based MOF, which was synthesized using 1,4-naphthalenedicarboxylic acid, scandium nitrate, oxidized pectin, and chitosan via a microwave-assisted method. Comprehensive characterization confirmed that the scandium-based MOF exhibits a high specific surface area, a crystalline porous structure, and functional groups responsible for adsorption and antibacterial activity. This study provides valuable insights for the design of highly efficient MOF-based flame retardants.

While the four articles included in this Research Topic represent only a small fraction of the extensive research in this field, they highlight the dynamic nature of studies on innovative flame-retardant materials, emphasizing the balance between efficiency, safety, and sustainability. Furthermore, a deeper understanding of the reaction and resistance to fire of these materials at the molecular level is essential. Fortunately, recent advancements in experimental techniques and theoretical models have opened up new avenues for addressing these challenges.

We sincerely thank all the authors for their outstanding contributions and for sharing their latest research findings. We also express our deep gratitude to the reviewers for their timely and thorough evaluations of the manuscripts, despite their busy schedules. Special thanks are extended to Mrs. Chen Wang, Journal Specialist and Assistant Editor of this issue, for her invaluable assistance, guidance, and secretarial support throughout the editorial process.
